# Comparing the transcriptomes of embryos from domesticated and wild Atlantic salmon (*Salmo salar* L.) stocks and examining factors that influence heritability of gene expression

**DOI:** 10.1186/s12711-016-0200-6

**Published:** 2016-03-17

**Authors:** Beatrix Bicskei, John B. Taggart, Kevin A. Glover, James E. Bron

**Affiliations:** Institute of Aquaculture, School of Natural Sciences, University of Stirling, Stirling, FK9 4LA UK; Institute of Marine Research, Bergen, Norway; Department of Biology, University of Bergen, Bergen, Norway

## Abstract

**Background:**

Due to selective breeding, domesticated and wild Atlantic salmon are genetically diverged, which raises concerns about farmed escapees having the potential to alter the genetic composition of wild populations and thereby disrupting local adaptation. Documenting transcriptional differences between wild and domesticated stocks under controlled conditions is one way to explore the consequences of domestication and selection. We compared the transcriptomes of wild and domesticated Atlantic salmon embryos, by using a custom 44k oligonucleotide microarray to identify perturbed gene pathways between the two stocks, and to document the inheritance patterns of differentially-expressed genes by examining gene expression in their reciprocal hybrids.

**Results:**

Data from 24 array interrogations were analysed: four reciprocal cross types (W♀ × W♂, D♀ × W♂; W♀ × D♂, D♀ × D♂) × six biological replicates. A common set of 31,491 features on the microarrays passed quality control, of which about 62 % were assigned a KEGG Orthology number. A total of 6037 distinct genes were identified for gene-set enrichment/pathway analysis. The most highly enriched functional groups that were perturbed between the two stocks were *cellular signalling* and *immune system*, *ribosome* and *RNA transport*, and *focal adhesion* and *gap junction* pathways, relating to *cell communication* and *cell adhesion molecules*. Most transcripts that were differentially expressed between the stocks were governed by additive gene interaction (33 to 42 %). Maternal dominance and over-dominance were also prevalent modes of inheritance, with no convincing evidence for a stock effect.

**Conclusions:**

Our data indicate that even at this relatively early developmental stage, transcriptional differences exist between the two stocks and affect pathways that are relevant to wild versus domesticated environments. Many of the identified differentially perturbed pathways are involved in organogenesis, which is expected to be an active process at the eyed egg stage. The dominant effects are more largely due to the maternal line than to the origin of the stock. This finding is particularly relevant in the context of potential introgression between farmed and wild fish, since female escapees tend to have a higher spawning success rate compared to males.

**Electronic supplementary material:**

The online version of this article (doi:10.1186/s12711-016-0200-6) contains supplementary material, which is available to authorized users.

## Background

Atlantic salmon (*Salmo salar* L.) has been subject to domestication, including directional selection for economically important traits, since the aquaculture industry was first established in the early 1970s [1, 2]. These breeding programs, which now extend beyond 10 to 12 generations, have been very successful. For example, selection for growth rate, which was the primary target of all Atlantic salmon breeding programs, resulted in farmed fish that reach a body size that is 2 to 3 times larger than that of wild fish when reared under identical farming conditions [3–6]. However, economically important traits may not be beneficial under wild conditions, for example offspring survival is reduced in farmed salmon compared to the wild parental lines under natural conditions [7–10]. Given the magnitude of the phenotypic and genotypic differences between wild and farmed salmon, it is feasible to investigate how domestication in general, and selection for specific traits, have altered both the structure and expression of the Atlantic salmon genome.

The early stages of the life cycle of Atlantic salmon involve (1) hatching of eggs that are deposited in the gravel of rivers, (2) nesting of the sac fry in the gravel and feeding on their yolk-sac, (3) emergence of the sac fry from the gravel, which is a process known as swim-up, and finally (4) transition from endogenous to exogenous feeding. These critical developmental stages that are associated with high mortality play a major role in shaping the evolutionary trajectory of the individual and the population in general [9, 11, 12]. While numerous studies have investigated genetic differences between farmed and wild salmon, to date, relatively few studies have specifically focused on the critical early-life stages. Exceptions include studies on fertilization success rate [13], speed of embryonic development and growth prior to exogenous feeding [14–16], mortality in the wild [17], and gene transcription e.g. [18–20].

During the first phase of development, before the maternal-to-zygotic transition activates zygotic transcription, the embryo almost exclusively relies on maternal mRNA and proteins [21], and until the initiation of exogenous feeding, pre- and post-hatching embryos depend largely on maternally deposited yolk for energy provision [22]. Generally, the success rate of eggs from farmed fish is lower than that of eggs from wild fish, due to nominally suboptimal maternal resources [23], but these differences vary across species and time and can be reduced by improving fish husbandry, feed formulation and rearing conditions [24]. For example, the Atlantic cod aquaculture industry has yet to achieve optimal farming practices since the success rates of fertilization and hatching of eggs from farmed broodstock are significantly lower than those from wild broodstock [25]. In contrast, recent common garden studies have reported largely comparable fertilization (in vitro [13] and in-vivo [26]) and hatching success rates [16] between wild and domesticated Atlantic salmon stocks. The few detected differences were in egg size (which is indirectly affected by maternal body size) and hatching rate [16, 26]; these two factors are considered to be interlinked and to differ between any two given populations [27]. Although variability of these traits may affect success rate under natural conditions [9, 12], these parameters are not used, per se, to discriminate “high” from “low” quality eggs and embryos [23].

Salmonid maternal effects have been thoroughly investigated for easy-to-measure phenotypic traits, such as egg and fry size, which have a significant impact on early survival [9, 11, 12]. However, studies at the transcriptional level are scarce. Debes et al. [15] emphasized the fact that multi-generational genetic studies on salmonids rarely use reciprocal hybrids due to logistical constraints. Even when reciprocal hybrids are used, data are often averaged across hybrids, which tends to hide maternal effects. A previous study that explored transcriptional differences in the early stages of development between farmed and wild Atlantic salmon strains included only non-reciprocal hybrids that were generated by fertilizing domesticated eggs with wild milt [20]. Although this study documented dominant inheritance patterns in the F1 hybrids, the lack of fully reciprocal pedigrees precluded a further analysis of its primary source, i.e. domestication and/or maternal effects.

With the decreasing cost of broad-scale gene expression studies, transcriptomic profiling of fish embryos is starting to receive increased attention. Recently, researchers have started to investigate how gene expression varies during embryonic development [28–31] and have attempted to identify transcripts and markers associated with embryo quality [31, 32]. Renaut et al. [33] showed that gene expression in hybrid embryos is affected when divergent populations are crossed. In our study, we used a custom oligo-microarray as a tool to identify genes and gene pathways that display differential expression between embryos from wild and domesticated Atlantic salmon stocks that were reared under identical conditions. By including reciprocal hybrids in the experimental design, heritability patterns were assessed to specifically explore the relative importance of maternal versus domestication effects on embryonic gene expression.

## Methods

### Biological samples

This study used experimental crosses between (1) the domesticated Norwegian Mowi strain, which has been under directional selection for at least ten generations and for a range of economically important traits, and (2) wild brood fish that were collected from the River Figgjo in the south west of Norway. Numerous studies have investigated the characteristics of the Mowi strain [4–6, 9, 20, 34], and both strains are described in detail in [20].

The experiment was initiated on November 23, 2011 when gametes were stripped from four domesticated (Mowi) and four wild (Figgjo) salmon. Two independent sets of reciprocal crosses were established, each set using gametes from a pair of domesticated (D) and wild (W) parents to create four family combinations (i.e. pure wild, W × W; pure domesticated, D × D; and reciprocal hybrids W♀ × D♂ and D♀ × W♂). Fertilized eggs from each of the eight families were placed into individual family hatching trays under identical conditions. On February 2 2012, (approximately 410 days post-fertilisation), eyed ova from each family (n = 30) were sampled, transferred to RNA stabilisation buffer i.e. RNAlater (3.6 M ammonium sulfate, 18 mM sodium citrate, 15 mM EDTA, pH 5.2) and immediately pierced with a 25G syringe needle for rapid penetration of the preservative. After overnight incubation at 8 °C, the RNAlater solution was drained and the eggs were stored at −70 °C until RNA extraction.

The experiment was conducted in accordance with Norwegian regulations for the use of animals in research. No specific permit was required for this experiment because embryos were sampled prior to hatching.

### RNA extraction and purification

**Fig. 1:**
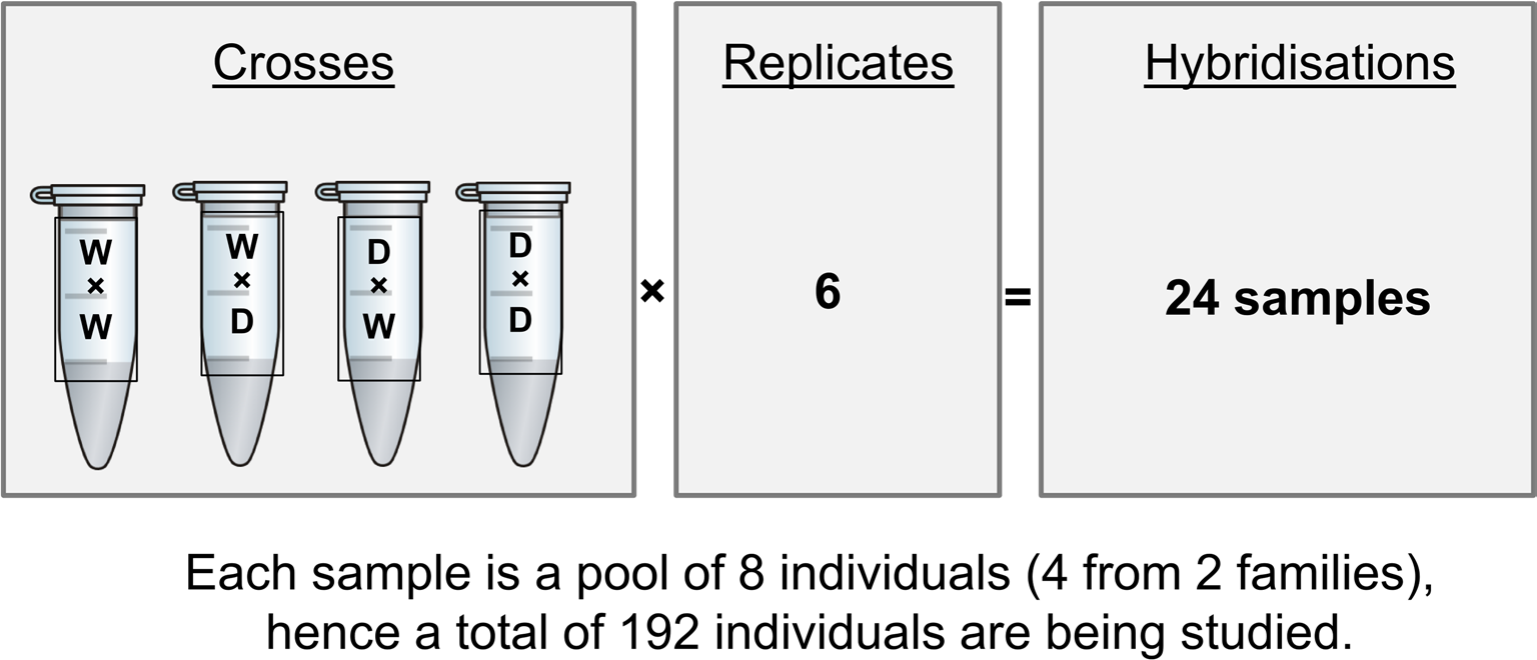
Schematic representation of the experimental design

Individual eyed eggs were homogenised in 1 mL Tri Reagent (Sigma-Aldrich^®^) using a Mini-Beadbeater-24 (BioSpec Products Inc.) and RNA was extracted following the manufacturer’s standard Tri Reagent protocol. RNA quantity and quality of individual embryos were assessed by spectrophotometry (NanoDrop ND-1000) and agarose gel electrophoresis, respectively. Each extraction yielded about 40 to 50 µg RNA. For each hybridisation sample (biological replicate), equal amounts of total RNA from eight individuals (four per family × two families) were pooled per reciprocal cross type (WW, DD, DW or WD) and then re-quantified and quality-assessed as described above (Fig. 1).

### Microarray experimental design

Microarray analysis was performed using a custom oligonucleotide microarray platform (Agilent) that included 4 × 44 K probes per slide (Salar3; ArrayExpress Accession number A-MEXP-2400). The general design of the microarray is described in [35] and was validated in subsequent studies e.g. [20, 35–38].

Dual-label hybridisations were undertaken, i.e. each experimental sample (Cy3-labelled) was competitively hybridised against a pooled reference control (Cy5-labelled) that included equimolar amounts from each experimental RNA sample. Thus, each experimental sample was assessed relative to a single common sample, which allowed a full range of comparisons between different states. The interrogations involved 24 separate hybridisations i.e. four reciprocal cross types (W♀ × W♂, D♀ × W♂; W♀ × D♂, D♀ × D♂) × six biological replicates (each replicate containing RNA from eight different individuals; four each from two families) (Fig. 1).

### RNA amplification and labelling

RNA from each biological replicate (pool of eight individuals) was amplified (TargetAmp™ 1-Round Aminoallyl-aRNA Amplification Kit, Epicentre Technologies Corporation) according to the manufacturer’s instructions. Following quality control (Nanodrop quantification and agarose gel electrophoresis), amplified RNA fragments (aRNA) were indirectly fluorescently labelled and purified. Briefly, dye suspensions (Cy3 and Cy5) in sufficient quantity for all labelling reactions were prepared by adding 42 µL of high-purity dimethyl sulfoxide (Stratagene) per tube of Cy dye (PA23001 or PA25001; GE HealthCare). Individual amplified samples (2.5 µL aRNA in 10.5 µL H_2_O) were denatured at 75 °C for 5 min, and then 3 µL 0.5 M NaHCO_3_ at pH 8.5 and 1.5 µL Cy3 dye were added. The common reference pool was labelled in the same way, but it was prepared in a single large-scale reaction i.e. 50 µg of pooled aRNA in 210 µL H_2_O were heat-denatured and then 60 µL 0.5 M NaHCO_3_ at pH8.5 and 20 µL Cy5 dye were added. All samples were incubated for 1 h at 25 °C in the dark, and purified through Illustra AutoSeq G-50 Dye Terminator columns (Qiagen). Dye incorporation and purity of all reactions were assessed spectrophotometrically (NanoDrop) and the products were also visualised on a fluorescent scanner (Typhoon Trio, GE Healthcare).

### Microarray hybridisation and quality filtering

All hybridisations were performed at the same time using the Agilent Gene Expression Hybridisation Reagent Kit (Agilent Technologies) according to the manufacturer’s instructions. For each reaction, 825 ng of Cy5-labelled RNA reference pool and 825 ng of a Cy3-labelled RNA test sample were combined in 35 µL of H_2_O to which 20 µL fragmentation master mix were added (11 µL of 10 × blocking agent, 2 µL 25× fragmentation buffer and 7 µL H_2_O). The reactions were then incubated at 60 °C in the dark for 30 min, chilled on ice, and mixed with 55 µL of 2× GEx hybridisation buffer (pre heated at 37 °C). Following centrifugation (18,000*g* for 1 min), the samples were kept on ice until they were loaded (103 µL) onto the microarray slides (four arrays per slide). Samples from the six biological replicates were distributed across different slides. Hybridisation was carried out in a rotating rack oven (Agilent Technologies) at 65 °C, 10 rpm for 17 h.

Following hybridisation, the microarray slides were washed in Easy-DipTM slide staining containers (Canemco Inc.). First, a 1-min incubation at room temperature (approximately 20 °C) in Wash Buffer 1 was performed, with gentle shaking at 150 rpm (Stuart Orbital Incubator). Slides were then briefly dipped into Wash Buffer 1 pre-heated at 31 °C and placed into Wash Buffer 2 (31 °C) for 1 min with gentle shaking at 150 rpm. Finally, the slides were transferred to acetonitrile for 10 s and finally incubated in the stabilization and drying solution (Agilent) during 30 s. The slides were then air dried and scanned within 3 h.

Slides were scanned at 5 μm resolution on an Axon GenePix Pro scanner at 70 % laser power. The “auto PMT” function was set to adjust PMT for each channel such that <0.05 % of the features were saturated and the mean intensity ratio of the Cy3:Cy5 signals was close to 1. We used the Agilent Feature Extraction Software (v 9.5) to identify features and extract background subtracted raw intensity values that were then transferred to the GeneSpring GX (version 13) software [39] to perform quality filtering and normalisation steps. Intensity values less than 1 were adjusted to 1 and a Lowess normalisation was carried out. Stringent quality filtering ensured that features that represented technical controls, saturated probes, probe population outliers or probes which were not significantly different from the background (based on a two-sided *t* test implemented in the Feature Extraction software) were removed. Finally, probes were retained if they were positive and significant in at least 75 % of the arrays in any two of the experimental groups. As a result, 31,491 probes passed quality control and were analysed further.

Details of the microarray experiment were submitted to ArrayExpress under accession number E-MTAB-3677. The recording of the microarray experimental metadata complies with the MIAME (minimum information about a microarray experiment guidelines).

### Microarray data analysis

Statistical analysis (T test and ANOVA) was performed by using the GeneSpring software (version 13), whereas the R software [40] was used for functional analysis (GAGE) and preparing graphs. Details of each analysis are provided below. To minimize repeat counting of the same gene, only transcripts that had BLAST [41] and/or KEGG annotations [42] were considered in the downstream analysis, and when multiple probes were present for the same gene, the probe with the lowest *p* value was chosen.

Functional analysis of the genetic differences between the offspring of wild and domesticated pure stocks was performed via the *gage* function of the GAGE (generally applicable gene-set/pathway analysis) package [43]. Gene-set tests establish correlations between functional groups and phenotype by detecting small but coordinated changes in gene expression [43]. Pairwise comparisons between replicates from domesticated fish embryos versus the average values for wild fish (‘1ongroup’ comparison) were performed and, as generally applied, results were considered significant if the corrected *p* value was >0.1. For ease of visualization and a more focused interpretation, pathways that were perturbed in both directions (2d) i.e. transcripts that were not restricted in terms of their direction of change, were further filtered by applying a *p* value cut off of 0.02. For a default (*p* ≤ 0.1) 2d pathway list, see Table S1 (see Additional file 1: Table S1). Since pathways that belong to the human disease functional group are particularly difficult to interpret in fish, this group was excluded from the gene-enrichment analysis. Significant pathways were further explored using the *essGene* function [43] to identify key genes. We used the package ggplot2 [44] to graphically represent the transcripts that were included in significantly perturbed pathways i.e. that varied by more than one standard deviation (SD) from the mean of all transcripts and differed significantly between domesticated and wild strains (t test unpaired unequal variance, p ≤ 0.05). When transcripts were represented in multiple KEGG groups, they were assigned the function for which the largest number of gene associations was found in the complete list.

To identify differentially-expressed transcripts between embryos of domesticated and wild salmon stocks, we performed a T test (unpaired unequal variance, Benjamini–Hochberg multiple-testing correction, corrected p ≤ 0.05) and applied a fold change filter ≥1.25 (see Additional file 1: Table S2) for details. Following hierarchical clustering (Pearson correlation), expression profiles of unique differentially-expressed transcripts between the two stocks were visualized as heatmaps (*gplots* package [45]).

To explore the heritability of differentially-expressed genes between stocks, one-way ANOVA (unequal variance) was performed with an FDR of 10 % (Benjamini–Hochberg) and Student Newman–Keuls (SNK) post hoc analysis. Differentially-expressed transcripts were assigned to the following categories of heritability:

1. Maternal effect: differentially-expressed transcripts that were identified between W♀ × W♂ versus D♀ × W♂ or D♀ × D♂ or W♀ × D♂;
2. Paternal effect: differentially-expressed transcripts that were identified between W♀ × W♂ versus W♀ × D♂ or D♀ × D♂ or D♀ × W♂;
3. Parental effect: differentially-expressed transcripts that were influenced by both maternal and paternal effects;
4. Maternal effect only: differentially-expressed transcripts that were influenced by maternal effects only;
5. Paternal effect only: differentially-expressed transcripts that were influenced by paternal effects only.

For normalised intensity values (*ni*) of the unique differentially-expressed genes: α = additivity = (W_ni_ − D_ni_)/2 and δ = dominance = ((W_ni_ + D_ni_)/2) − hybrid_ni_ were calculated. The values for α and δ/α were plotted using the *ggplot2* package [44]. A transcript with a level of gene expression in the hybrid that was midway between that for the parents had an additive effect (perfect additivity: δ/α = 0). A transcript with a level of gene expression in the hybrid that was close to that of one of the two parents had rather a dominant effect (domesticated dominance, δ/α = 1; wild dominance, δ/α = −1). Group memberships were assigned as follows by dividing the intervals into two parts:

- additivity, if −0.5 < δ/α < 0.5;
- wild dominance, if −1.5 < δ/α < −0.5;
- domesticated dominance, if 0.5 < δ/α < 1.5;
- over-dominance, if δ/α fell outside the interval between −1.5 and 1.5.

For ease of interpretation of the plots, genes with a δ/α above 5 were excluded from the scatter graph but were considered in the table on heritabilities.

## Results

### Functional analysis

**Fig. 2:**
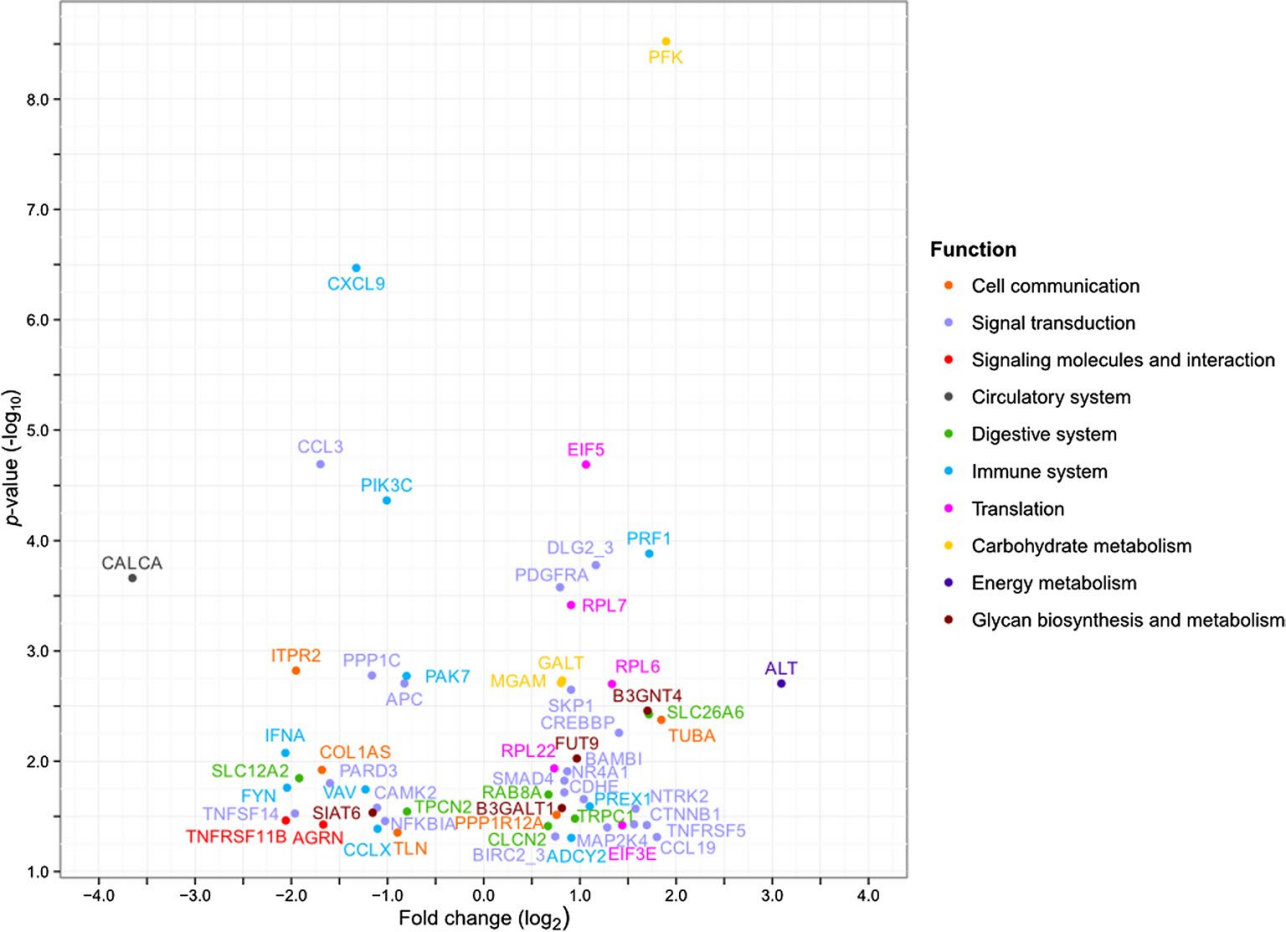
Key genes of the perturbed pathways. Differentially-expressed genes (T test *p* ≤ 0.05) between wild and domesticated embryos and identified as essential for the pathways perturbed between pure stocks (Table 1). Genes are plotted according to log_2_ fold change (domesticated vs. wild) and −log_10_ *p* value (T test), and *color-coded* by biological function. The list of plotted genes and values are included in Table S4 (see Additional file 1: Table S4)

For the functional analysis, KEGG annotation (KO) was used. Approximately 62 % of the probes that passed quality filtering were assigned KO numbers and about 31 % of these returned unique annotations. Hence, 6037 genes were included in the gene-set enrichment analysis, which revealed a range of pathways with significant differential gene expression between embryos of wild and domesticated salmon (Table 1). The *ECM*-*receptor interactions* pathway was down-regulated in the domesticated fish embryos compared to the wild fish embryos, whereas pathways that are involved in *genetic information processing* and *metabolism* functions were up-regulated. *Genetic information processing*-related pathways play a role in *mRNA translation*, whereas *metabolism*-related pathways are associated with *carbohydrate*, *lipid* and *energy metabolism*. In addition, the most significant two-way perturbed pathways were related to *environmental information processing*; *cell signalling*, in particular, and *organismal systems*; including *digestive*, *immune* and *nervous systems*. Most differentially-expressed transcripts and major contributors to these significant pathways were members of signal transduction pathways (Fig. 2). Other KEGG functional groups that displayed more than ten differentially-expressed genes included the *immune system*, *cell communication* and *signalling molecules and interaction.* There was considerable gene overlap between these groups, for details (see Additional file 1: Table S3).

**Table 1:** Differentially-expressed pathways in domesticated versus wild embryos

KEGG functional group	KEGG sub-group	KEGG pathway	*p* value	Direction of perturbation	Sac fry [20]	Feeding fry [20]
Cellular processes	Cell communication	Focal adhesion	0.00051	Two-way perturbed		
		Gap junction	0.00036	Two-way perturbed		
Environmental information processing	Signal transduction	Hippo signaling pathway	0.00040	Two-way perturbed	Up-regulated	
		MAPK signaling pathway	0.00101	Two-way perturbed		
		NF-kappa B signaling pathway	0.00021	Two-way perturbed	Down-regulated	Down-regulated
		Wnt signaling pathway	0.00213	Two-way perturbed	Up-regulated	
	Signaling molecules and interaction	Cell adhesion molecules (CAM)	0.00069/0.00144	Up-regulated/two-way perturbed		
		Cytokine-cytokine receptor interaction	<0.00001	Two-way perturbed	Down-regulated	
		Neuroactive ligand-receptor interaction	0.00001	Two-way perturbed	Two way perturbed	Down-regulated
		ECM-receptor interaction	0.00016	Down-regulated		Up-regulated
Organismal systems	Circulatory system	Vascular smooth muscle contraction	0.00032	Two-way perturbed		
	Development	Osteoclast differentiation	0.00019	Two-way perturbed	Two way perturbed	
	Digestive system	Mineral absorption	0.00011	Up-regulated		
		Pancreatic secretion	0.00164	Two-way perturbed		Up-regulated
		Salivary secretion	0.00117	Two-way perturbed		
	Endocrine system	GnRH signaling pathway	0.00014	Two-way perturbed		
	Immune system	Chemokine signaling pathway	0.00017	Two-way perturbed	Down-regulated	Down-regulated
		Fc epsilon RI signaling	0.00026	Two-way perturbed	Down-regulated	Down-regulated
		Natural killer cell mediated cytotoxicity	0.00004	Two-way perturbed		
		T cell receptor signaling pathway	0.00002	Two-way perturbed		
	Nervous system	Glutamatergic synapse	0.00154	Two-way perturbed	Down-regulated	
		Long-term potentiation	0.00001	Two-way perturbed		
Genetic information processing	Translation	Ribosome	0.00383	Up-regulated	Up-regulated	
		RNA transport	0.00174	Up-regulated	Up-regulated	
Metabolism	Carbohydrate metabolism	Fructose and mannose metabolism	0.00183	Up-regulated		
		Galactose metabolism	0.00168	Up-regulated		
	Energy metabolism	Carbon fixation in photosynthetic organisms	0.00494	Up-regulated		Up-regulated
	Glycan biosynthesis and metabolism	Glycosphingolipid biosynthesis—lacto and neolacto series	0.00316	Up-regulated		
	Lipid metabolism	Sphingolipid metabolism	0.00229	Up-regulated		

KEGG based functional representation of the pathways differentially perturbed between wild and domesticated embryos and their significance in a previous study conducted on sac and feeding fry

### Expression profiling

**Fig. 3:**
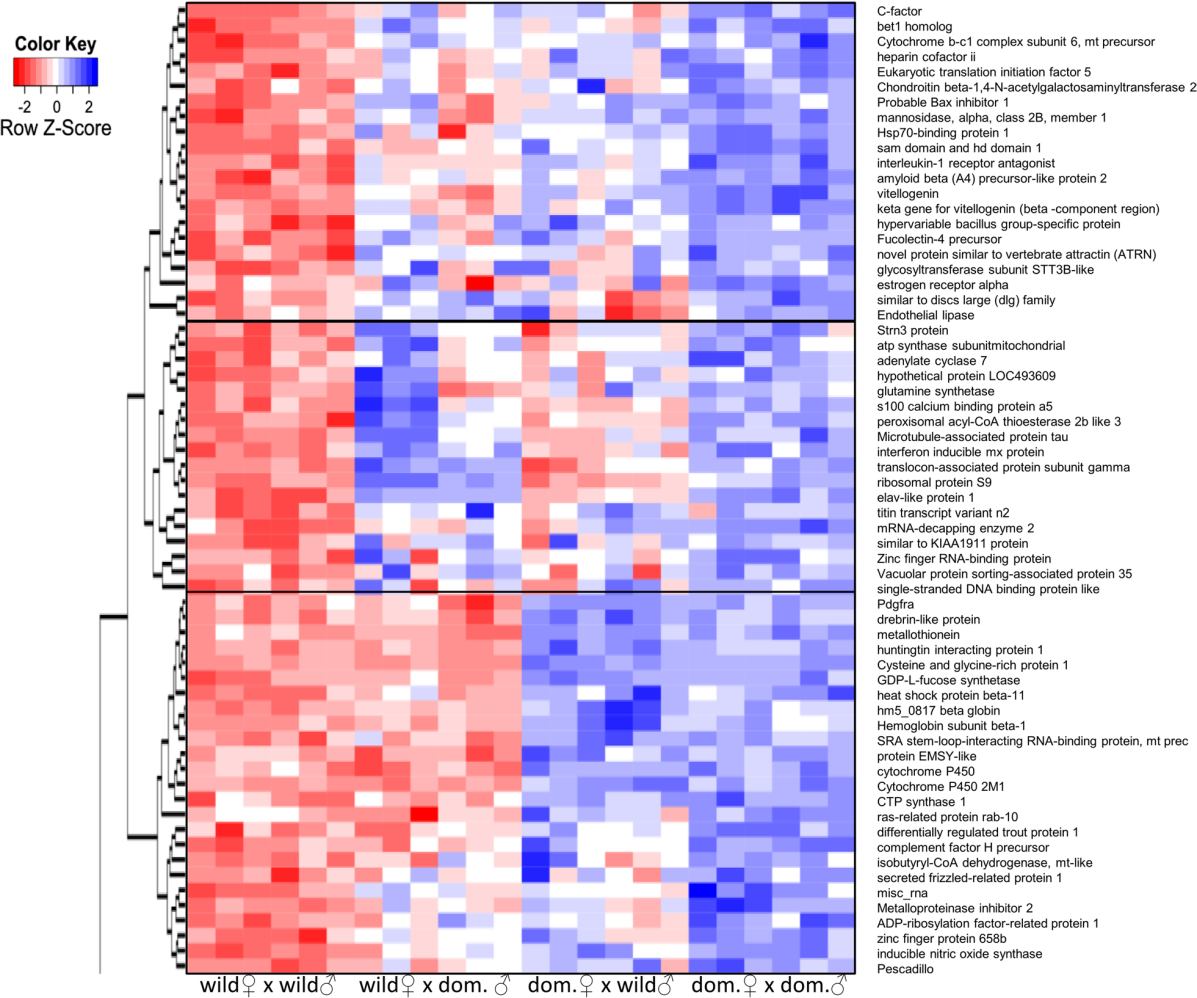
Up-regulated differentially expressed transcripts. Hierarchical clustering of the expression profiles of unique transcripts up-regulated in domesticated embryos compared to wild embryos

**Fig. 4:**
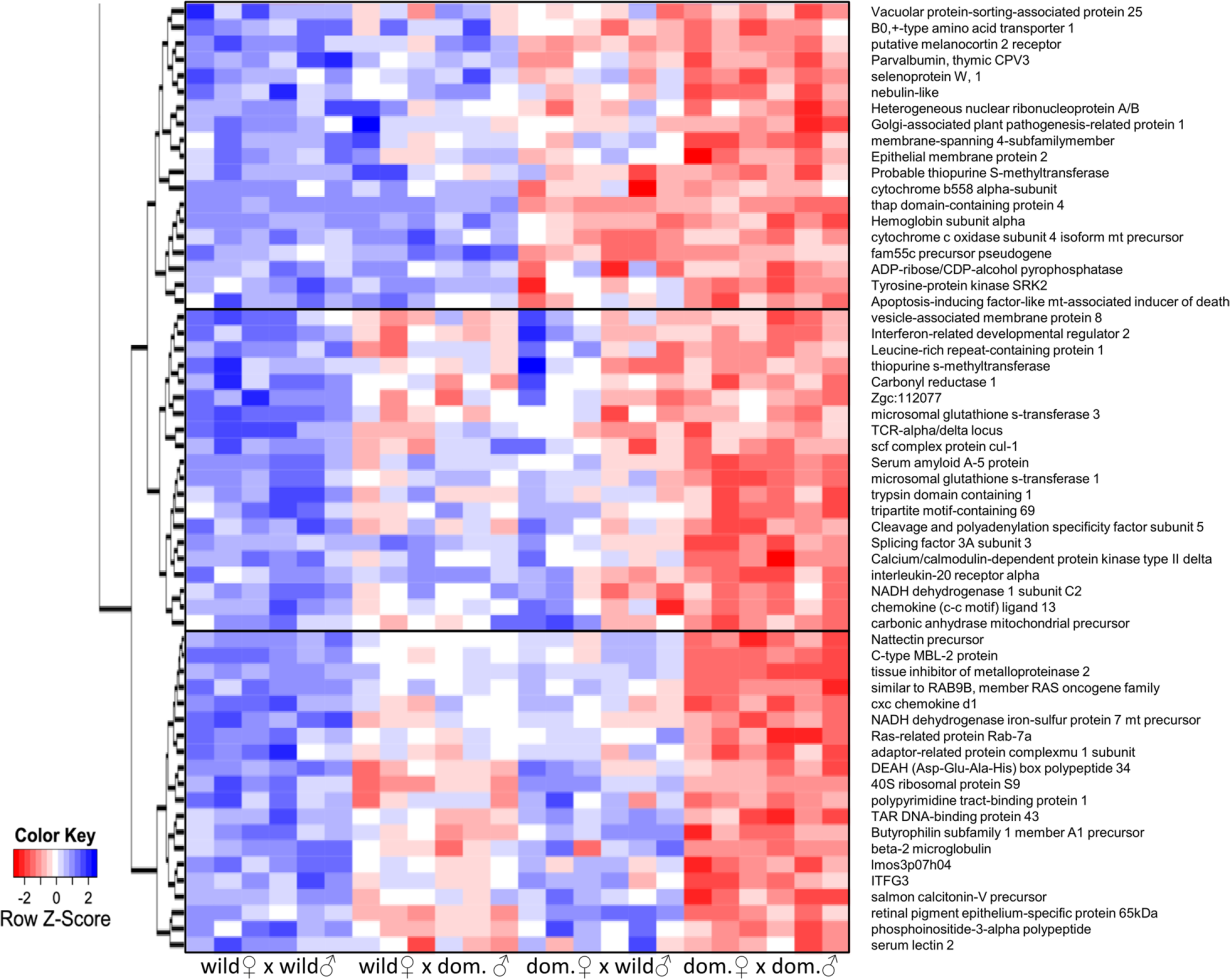
Down-regulated differentially expressed transcripts. Hierarchical clustering of the expression profiles of unique transcripts down-regulated in domesticated embryos compared to wild embryos

T tests identified 165 transcripts that showed significantly different gene expression levels between embryos of domesticated and wild salmon stocks and corresponded to 123 unique annotated transcripts. Hierarchical clustering of the differences in gene expression revealed both additive and dominant behaviours (Figs. 3, 4). The clusters with the most pronounced behaviour were related to maternal influence, such as the bottom cluster in Fig. 3 and the top cluster in Fig. 4, which both contain several *cytochrome*-related genes.

### Heritability analyses

**Fig. 5:**
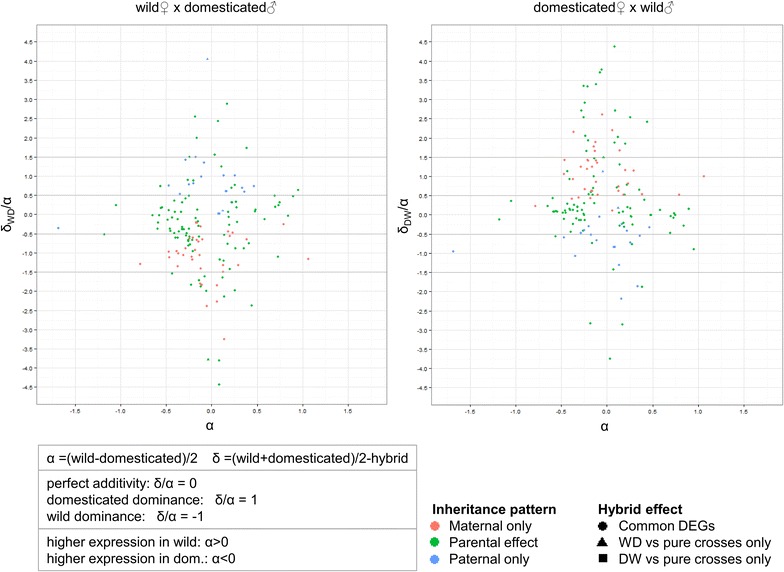
Heritability predictions of the differentially-expressed genes between the two hybrid stocks. *DEG* differentially-expressed gene, *WD* wild♀ × domesticated♂, *DW* domesticated♀ × wild♂

**Fig. 6:**
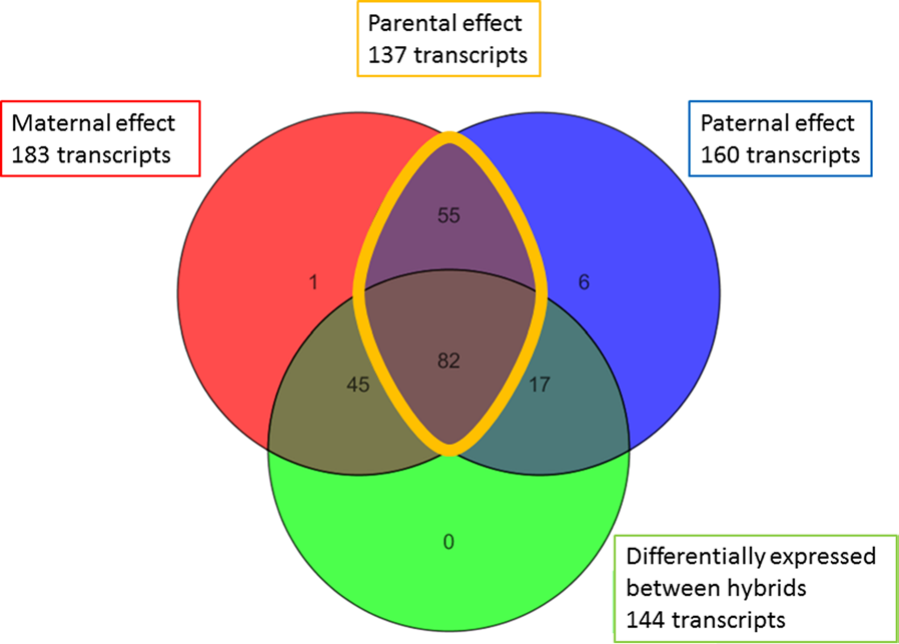
Number of transcripts differentially expressed between stocks and their inheritance pattern. Differences observed between hybrid and pure crosses are categorized as influenced by maternal, paternal or parental effects (see “Methods” for details). The number of differentially-expressed transcripts identified between hybrid crosses is also shown

To further investigate the significance of parental effects that were revealed by expression profiling, additive and dominance parameters were calculated and plotted (Table 2; Fig. 5). Among the 208 transcripts that showed differential expression between the four experimental groups by one-way ANOVA, only two were significantly different between the pure crosses and were not considered further. There were no observed differences between hybrid × hybrid crosses that were not also seen between hybrid × pure crosses (Fig. 6).

**Table 2:** Proportions of differentially-expressed genes that display various inheritance patterns

Life stage	Hybrid type	Unique genes	Wild overdominant (%)	Wild dominant (%)	Additive (%)	Domesticated dominant (%)	Domesticated overdominant (%)	Experiment
Embryo	wild♀ × domesticated♂	162	18.5	27.8	33.3	14.2	6.2	Current study
Embryo	domesticated♀ × wild♂	156	4.5	9.0	42.3	23.1	21.2	Current study
Fry	domesticated♀ × wild♂	25	0.0	0.0	48.0	52	0.0	[20]
Feeding fry	domesticated♀ × wild♂	313	1.6	6.1	45.0	42.2	5.1	[20]

Based on a heritability analysis of the differentially expressed genes and a comparison of the inheritance patterns to a previous study conducted in sac and feeding fry. For explanation of the various categories see the “Methods” section

The remaining 206 differentially-expressed transcripts corresponded to 165 unique genes that were further analysed. The vast majority of the differences (153 genes) were shared by both hybrid crosses, whereas nine and three additional genes were unique to either the W♀ × D♂ or D♀ × W♂ hybrids, respectively. For the reciprocal hybrids, most transcripts displayed either an intermediate level of expression (33.3 and 42.3 %) or dominance versus over-dominance (27.8 vs. 18.5 % and 23.1 vs. 21.2 %, for the reciprocal hybrids, respectively), which was in favour of a maternal effect (Table 2). However, W♀ × D♂ hybrids showed a stronger combined (wild or domesticated dominance) dominance effect (42 vs. 32.1 %) and a weaker additive effect (33.3 vs. 42.3 %) compared to D♀ × W♂ hybrids.

Since most of the transcriptomic differences detected between the two stocks were shared by both reciprocal hybrids, expression levels of the transcripts for the hybrids and the pure crosses were compared to determine whether these were primarily influenced by domestication or parental line factors. Visualisation of the dominance behaviour (Fig. 5) showed that most transcripts that were differentially expressed between the two stocks were either additive or maternally-dominant. For ease of visualization, 15 over-dominant genes were excluded from the scatterplot (Fig. 5), due to large δ/α.

## Discussion

In the literature, the first comparisons of genome-wide gene transcription of Atlantic salmon fry based on microarray data reported that five to seven generations of domestication and selection had induced heritable changes of gene expression in cultured relative to wild fish [18, 19]. Differences were observed in common pathways but did not necessarily involve the same genes within a given pathway, which is referred to as differences in ‘genetic architecture’ between stocks. A more recent study [20] demonstrated that whereas common differences could be observed between different life stages, some of the affected key pathways were stage-dependent. Since the experimental designs of these earlier studies included the analysis of D♀ × W♂ hybrids only, it was not possible to distinguish dominant parental effects from domestication effects.

Our study aimed at expanding existing knowledge on transcriptomic differences between wild and domesticated Atlantic salmon by investigating embryos for the first time, and using reciprocal hybrids to help dissect parental effects from the effects of domestication. Focusing on early-life stages has also the benefit of minimizing environmental effects and the impact of early-growth divergence on the transcriptome due to the fact that farmed salmon outgrow wild salmon by up to threefold by 4 months into feeding [5]. However, this approach has several inherent limitations. The microarray analysis is restricted to the set of preselected probes on the platform. Analysis of transcriptomes for whole embryos is likely to be relatively insensitive to differences in tissue-specific transcript expression, especially from smaller organs and low abundance cell types. For example, cellular signalling occurs in all cells regardless of the tissue of origin and, thus, the members of these pathways are expressed across all organs [46]. Organism-wide expression of cell signalling genes may provide support for the detection of this function when gene expression of whole individuals is studied. It is also important to bear in mind that since our study used only one wild and one domesticated salmon stock, some of the observed differences may be specific to these stocks and not necessarily reflect a domestication effect per se.

Overall, we identified pathways that are related with *metabolic*, *immune* and *nervous system*, *genetic* and *environmental information processing* functions for which altered gene expression was observed between the wild and domesticated Atlantic salmon embryos that were studied here. Our findings build on earlier studies of whole-animal transcriptomic responses to domestication in a number of fish species and for different life stages [18–20, 31, 32]. In the next section, we examine in more detail some of the key pathways that we identified, in order to set the observed differences in a biological context.

### Domestication is a form of adaptation

Domestication is possible because “organic beings” have the ability to adapt to the changing environment, which is imposed on them [47]. As such, one would expect that the biological pathways that are relevant to adaptation to a farm environment would be differentially expressed between wild and domesticated fish. *Cell signalling* mediates responses to internal and external environmental cues and therefore may be affected by domestication. *Cell signalling* is involved in the control of the basal level of cell replication, differentiation and apoptosis and the regulation of metabolic events, including the ability to receive signals and to respond to constantly altered physiological requirements. Such control is achieved through the action of three broad classes of signalling molecules: neurotransmitter substances, hormones and cytokines or growth factors [46]. In lower vertebrates, such as fish, cytokines and neuropeptides play roles both in the neuroendocrine and immune systems, including responses to stress [48, 49]. It has been suggested that the fish domestication process involves increased selection pressure on genes and pathways which contribute to improved tolerance to acute and chronic stress, since individuals that perform better under farm conditions are more likely to be selected for broodstock [5, 50]. As a key mediator of the stress response, modulation of cellular signalling is expected to play a role in the process of domestication as clearly shown in our study by the detection of differential expression of stress-associated nervous and endocrine pathways between the two fish stocks. In particular, we found that the *glutamatergic synapse* pathway differed between the two fish stocks, as previously reported between wild and domesticated Atlantic salmon fry [20]. Changes in this pathway are known to be associated with domestication in pigs and the expression of glutamate receptors that affect the neural control of eating behaviour is linked to tameness [51]. In addition, two other pathways that are linked with domestication in birds [52] were highlighted in our study, i.e. *long*-*term potentiation*, which has a role in memory consolidation [53] and *GnRH signaling*, which is a master regulator of vertebrate reproduction [54].

### Potential trade-off between immune function and growth

In addition to pathways that are involved in the adaptation to a farm environment, domestication may also affect pathways, which enhance farm traits that are important to broodstock selection, in particular increased growth.

#### Up-regulated mRNA translation

In addition to its role in the response to stress, the *mitogen activated protein kinase* (*MAPK*) *signalling* pathway also regulates *mRNA translation* and classical *MAPK signalling* promotes protein synthesis [55]. Thus, selection for improved growth traits in domesticated fish may explain why *MAPK signalling* pathways were enriched in our analysis. Previous studies have reported that *MAPK signalling* pathways are also involved in the domestication process of birds [52] and mammals [56–58]. Up-regulation of *ribosome* and *RNA transport* pathways in domesticated salmon embryos may also reflect processes which can enhance growth. Ribosomes are the site of protein synthesis, which is principally regulated at the initiation stage of translation, thus allowing plasticity of expression. The differential expression of *translation initiation factors 3E* and *5and large ribosomal subunits 6* and *7* that we identified in our study, are also involved in the regulation of protein synthesis [59]. Previous studies have reported that genes that affect protein synthesis, and hence growth, are over-represented in comparisons of expression levels of transcripts between wild and domesticated salmonid stocks [18, 20, 60–62]. By comparing fast- and slow-growing rainbow trout lines (*Oncorhynchus mykiss*), Xu et al. [29] concluded that up-regulation of genes that affect protein synthesis occurred at stages that corresponded to the earlier onsets of developmental processes in fast-growing families, i.e. as early as 15 days post-fertilisation.

#### Up-regulated metabolic pathways

During early development, the embryo relies on yolk sac-derived nutrients to sustain its growth and survival. These include yolk lipids, which are the source of essential fat-soluble vitamins and triacylglycerol, and cholesterol, which is a required component of cell signalling molecules, membrane components, and sources of fuel [63]. Many of the digestive functions that occur in hatched fry are known to be active in embryos [64], particularly after the eyeing stage as examined in the current study. Several pathways that impact *lipid, carbohydrate and energy metabolism* functions were up-regulated in the domesticated Atlantic salmon embryos compared to the wild embryos. These findings mirror those reported for feeding fry from the same two stocks [20], although the specific pathways differed. This possibly reflects differences in the processes for metabolizing yolk deposits and external food. For example, carbohydrate metabolism pathways that are differentially expressed between wild and domesticated embryos are involved in *fructose*, *mannose* and *galactosemetabolism*, whereas *glycolysis/gluconeogenesis* and *propanoate metabolism* pathways were identified in the feeding fry life-stage. *Sphingolipid metabolism* was detected as a differentially-expressed lipid metabolism pathway in the embryo stage, whereas *fatty acid degradation* and *elongation* and *glycerolipid metabolism* were differentially perturbed in the feeding fry stage. Indeed, activation of the glycolytic and fatty acid pathways is associated with gene expression changes that occur during the transition from endogenous to exogenous feeding of fish [65].

### Down-regulation of immune pathways

Cell signalling is particularly important during embryonic development [66] and the reciprocal gene regulation in both directions is characteristic of these regulatory pathways [43]. Major overlaps between members of signalling and immune pathways may mask the direction of change of immune pathways. For this reason, the expression of some key genes was investigated, including representatives of different groups of cytokines i.e.: four chemokines (*CCL* and *CXCL*), three *tumour necrosis factor* (TNF) *ligands/receptors*, and an *interferon α* (*IFN*-*α*). Most of these genes had a lower level of expression in domesticated embryos than in their wild counterparts. Chemokines and TNF play a pivotal role in immune function, but some members are also involved in stress responses and developmental processes [67–69]. It was previously suggested that domestication in salmonids may have resulted in immunosuppression, due to a trade-off between growth and immune functions [3]. In addition, since domesticated fish generally display a higher tolerance to stress, immune genes may have been collaterally selected during domestication [5, 50].

Two cytokines, i.e. *C*–*C motif chemokine 19* (*CCL19*) and *TNFR superfamily member 5* (*TNFRSF5*) are not down-regulated in domesticated versus wild embryo salmon, contrary to the behaviour expected based on the above argument. *CCL19* is referred to as a homeostatic or dual function chemokine [70] and has a role in the formation of the embryonic axis in zebrafish [71]. Hence, it may have a more important role in developmental functions than in immune functions. *TNFRSF5* does not play a role in any of the significantly differentially-expressed immune pathways and was detected only in signalling pathways. It should be noted that, *interferon regulatory factor 7* (*IRF7*), a transcription factor that is known to regulate *IFN*-*α genes* [72] and which, in our study, was down-regulated in the domesticated salmon embryos, has been proposed as a marker for assessing egg quality in Atlantic halibut (*Hippoglossus hippoglossus*) and is associated with hatching success [31].

### Organogenesis

Two *cell communication* pathways and the *cell adhesion molecules* pathways were differentially expressed between wild and domesticated Atlantic salmon embryos but not in yolk-sac fry or feeding fry [20], which probably reflects life-stage specific differences between stocks. These and several other differentially-expressed signalling pathways identified in our study (but not necessarily unique to embryos), are all known to participate in organ development. For example, the *Hippo signalling* pathway, which was differentially expressed between wild and domesticated strains, is involved in determining organ size and mediates crosstalk between other pathways [73]. NF-KB/IKB proteins, in addition to their immune function, are vital for organogenesis, e.g. zebrafish notochord development [74]. The *wnt signalling* pathway which is responsible for tissue morphogenesis, is up-regulated in domesticated Atlantic salmon sac fry [20]. According to Steinberg’s differential adhesion hypothesis, the basis of organ self-assembly is the segregation of cells with similar adhesive properties to achieve the most thermodynamically-stable pattern [75]. Thus, WNT proteins and cellular communication/cell adhesion pathways are closely linked [76] and we found that they were differentially expressed between wild and domesticated Atlantic salmon embryos. Sphingolipids, and their more complex, glycosylated derivatives, glycosphingolipids, as well as being components of cell membranes are also involved in cell signalling and adhesion [77]. In line with this, *glycan* and *lipid metabolism* pathways were up-regulated in the domesticated salmon embryos. The epithelial–mesenchymal transition (EMT) is a process during which tightly adjoined basal polarity epithelial cells acquire migratory mesenchymal properties [78]. This process involves most of the differentially-expressed *signalling* and *cellular communication* pathways identified in our study, including *MAPK, NF*-*kappa B, and wnt signalling, cytokine*–*cytokine receptor interactions, ECM*-*receptor interactions, cell and focal adhesion and gap junction.* The role of the epithelial–mesenchymal transition during development has an effect on organ development and neural crest cell migration [79]. Although changes that occur during neural crest development through domestication may provide an explanation for some of the shared similarities between domesticated species [80], its role in organ development fits the sampling timeline better. Sampling took place after eyeing of embryos, which occurs in the last third of embryogenesis. This phase of development is characterized by organogenesis, with the appearance of fins and the formation of the internal organs and circulatory system. Eyeing occurs in stage 24 of the development of salmons, whereas the neural tube is considered to be formed by stage 14 [64, 81].

### Parental effects on gene expression

For genes that are significantly differentially expressed between pure crosses, gene expression in the hybrids ranged from intermediate to fully polarized expression towards one or the other parent. Hierarchical clustering revealed that the behaviour of a number of genes in the hybrids reflected that of the maternal parent (wild or farmed). Within this group, there was a high abundance of *cytochrome*-related genes, which are involved in oxidative phosphorylation (*mitochondrial subunit/precursors of the cytochrome b*-*c1 complex subunit 6* and *cytochrome c oxidase subunit 4 isoform*, *NADH dehydrogenase 1 subunit C2* and *NADH dehydrogenase iron*-*sulfur protein 7* and an *ATP synthase*) and the metabolism of xenobiotics (*microsomal glutathione s*-*transferase 1* and *3* and *cytochrome P450, family 2, subfamily D*). Previous studies have reported that these processes have been affected by domestication in a number of fish species including brook charr (*Salvelinus fontinalis*), Atlantic salmon and Atlantic cod (*Gadus morhua*) [32, 61, 82]. Crockford [83–85] proposed that domestication is the product of heterochrony, i.e. changes in developmental rates and/or timing that are induced by thyroid hormone-induced oxidative reaction and metabolism rates, to which carbohydrates and lipids contribute. Two haemoglobin subunits were also differentially regulated between wild and domesticated Atlantic salmon embryos and clustered with genes that showed maternal influence (Figs. 2, 3). Previously, haemoglobin genes were also shown to be differentially regulated between multiple wild and domesticated brook charr reciprocal hybrids, which suggests consistent parental effects [86].

Maternal effects are known to be particularly frequent during the embryonic stage of fish [21, 22] but there is also a growing body of evidence for paternal effects [87]. In our study, most of the transcripts that were differentially expressed between a hybrid and a pure cross were common to both reciprocals. These shared differences were more likely to show dominance with respect to the origin of the mother rather than the origin of the stock, which indicates maternal dominance. We observed that differential expression of wild♀ × domesticated♂ hybrids showed a slightly higher combined dominance (42 vs. 32.1 %) and lower additivity (33.3 vs. 42.3 %) than domesticated♀ × wild♂ hybrids. In line with these results, Bougas et al. [88] highlighted the relevance of additivity (54.3 %) and the importance of maternal effects (40 %) when comparing the inheritance of gene expression of wild-domesticated brook charr hybrids.

### Implications for interactions between wild and farmed salmonids

Fish escaping from commercial farms and subsequent genetic interactions with wild conspecifics represent a major environmental challenge to a sustainable Atlantic salmon aquaculture industry [89]. Each year, hundreds of thousands of farmed Atlantic salmon escape into the wild. Although many of these remain unaccounted for, a significant number do enter rivers [90, 91] and interbreeding between wild and farmed salmon that lead to genetic changes of the wild populations has been reported in Ireland and Norway [92–96]. This has caused significant international concern over the long-term fitness of wild populations, given that wild salmon populations may display local adaptations to the rivers they inhabit [97], and that the offspring of farmed salmon show reduced survival in the wild compared to the offspring of wild salmon [7–9, 98]. The transcriptomic differences that were identified in our study may reflect the influence of different selection pressures acting on wild and domesticated Atlantic salmon populations. Adaptation to a farm environment is unlikely to be advantageous under natural conditions. The high prevalence of maternal effects is of particular concern, since domesticated females are more likely to contribute to gene flow from farm escapees than males [99].

## Conclusions

Our findings document the differential expression of gene pathways between Atlantic salmon eyed embryos of wild and domesticated origin, which were fertilised and incubated under identical conditions. The data indicate that even at this early developmental stage, transcriptional differences between the stocks exist and affect pathways that are relevant to wild and domesticated environments. By analysing the data from reciprocal hybrids, the potential significance of maternal effects in wild × domesticated hybrids and the relatively high percentage of over-dominant gene expression, which may be typical of the embryo stage, were highlighted. In order to draw more general conclusions regarding the outcome of the genetic interactions between wild and domesticated fish, more evidence is required from future studies on multiple strains, rather than single strains as was the case here.

## Additional file

10.1186/s12711-016-0200-6 List of differentially-expressed 2d pathways. The complete list of differentially-expressed 2d pathways that were identified by an adjusted p ≤ 0.1. **Table S2.** List of differentially-expressed transcripts. Details of the transcripts identified as differentially expressed between wild and domesticated embryos. **Table S3.** Essential genes of the differentially-expressed pathways. Gene members of pathways that contribute most to their differential expression. **Table S4.** Key genes of the differentially-expressed pathways.
